# Alisol F 24 Acetate Enhances Chemosensitivity and Apoptosis of MCF-7/DOX Cells by Inhibiting P-Glycoprotein-Mediated Drug Efflux

**DOI:** 10.3390/molecules21020183

**Published:** 2016-02-04

**Authors:** Guixiang Pan, Tingting Li, Qingqing Zeng, Xiaoming Wang, Yan Zhu

**Affiliations:** 1 Tianjin State Key Laboratory of Modern Chinese Medicine, Tianjin University of Traditional Chinese Medicine, Tianjin 300193, China; guixiangp@163.com (G.P.); litingting2559@163.com (T.L.); zengqing20080629@126.com (Q.Z.); xiaoming_w@yeah.net (X.W.); 2 Tianjin International Joint Academy of Biomedicine, Tianjin 300457, China

**Keywords:** alisol F 24-acetate (ALI), multidrug resistance (MDR), doxorubicin, P-glycoprotein (P-gp)

## Abstract

Multidrug resistance (MDR) is a prime reason for numerous failed oncotherapy approaches. In the present study, we investigated whether Alisol F 24 acetate (ALI) could reverse the MDR of MCF-7/DOX cells, a multidrug-resistant human breast cancer cell line. We found that ALI was a potent P-glycoprotein (P-gp) inhibitor, in the Caco-2-monolayer cell model. ALI showed a significant and concentration-dependent cytotoxic effect on MCF-7/DOX cells in combination with doxorubicin by increasing intracellular accumulation and inducing nuclear migration of doxorubicin. However, ALI had no such effect on MCF-7 cells. In addition, ALI also promoted doxorubicin-induced early apoptosis of MCF-7/DOX cells in a time-dependent manner. These results suggest that ALI can enhance chemosensitivity of doxorubicin and reinforce its anti-cancer effect by increasing its uptake, especially inducing its nuclear accumulation in MCF-7/DOX cells. Therefore, ALI could be developed as a potential MDR-reversing agent in cancer chemotherapy in further study.

## 1. Introduction

Currently, one challenge faced by cancer chemotherapy is the development of multidrug resistance (MDR) [1]. After a long-term exposure to anticancer drugs in malignant tumor cells, MDR cells acquire resistance to one chemotherapeutic drug, and and may also become resistant to some other anticancer drugs featuring different structures and functions [2]. A wide range of structurally diverse drugs used to treat cancer are extruded from cells, including doxorubicin, vinblastine, epipodophyllotoxins and paclitaxel [3]. Efflux proteins that mediate MDR of cancer cells are mainly the superfamily of adenosine triphosphate binding cassette-(ABC) transporters, including P-glycoprotein (P-gp) [4], multi-drug resistance protein (MRP) [5] and breast cancer resistance protein (BCRP) [6,7]. The overexpression of P-gp is considered to be a classic mechanism of MDR [8]. Encoded by ABCB1 gene, P-gp is a transmembrane protein and works as an ATP-dependent drug transporter. P-gp increases efflux of anticancer drugs, reducing intracellular drug levels and causing consequent drug insensitivity [9,10]. Apart from the development of multidrug resistance, severe side effects also stand in the way of cancer chemotherapy. The calcium channel blocker verapamil and the immune suppressor cyclosporin A have been proposed as modulators of P-gp to be used in association with anticancer drugs substrates of this efflux pump. However, serious cardiovascular adverse reactions, immune suppression and kidney toxicity have emerged [11,12].

In our paper, a new P-gp inhibitor was combined with doxorubicin in an effort to reverse MDR. Natural products serve as a reliable and excellent source for pharmaceutical development in recent years. According to the U.S. National Cancer Institute, it is convincingly demonstrated that nearly 69% of all anticancer drugs approved from the 1980s to 2002 are either natural products or derivatives synthesized based on the information provided by natural products [13]. A variety of natural product-derived drugs have been demonstrated to show reversal effects on MDR cells, including moollugin [14], guggulsterone [15] and rabdosia rubescens [16]. They enhance chemosensitivity of MDR-cells by increasing cellular influx of anticancer drugs. Meanwhile they are less toxic than chemically synthesized substances. Paclitaxel, a natural product from the bark of the Pacific yew *Taxus brevifolia*, reveals curative effects on human breast cancer [17]. Therefore, natural products from traditional Chinese medicines (TCM) hold a great promise for discovering safer and more efficacious MDR-reversal agents.

In China, *Rhizoma alismatis* is used to clear damp and heat as well as to promote diuresis. In recent years, *Rhizoma alismatis* has achieved initial success in exerting obvious effects on diuretic, anti-inflammatory hypoglycemic, hypolipidemic and antihypertensive therapies, inhibiting formation of kidney stones and regulating immune function [18].

**Figure 1:**
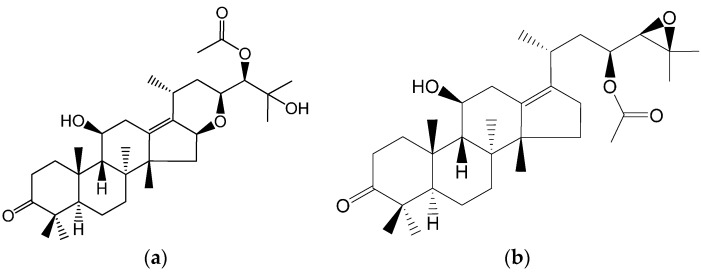
Chemical structures of alisol F 24-acetate (**a**) and alisol B 23-acetate (**b**).

Alisol F 24 acetate (ALI) is a triterpene (Figure 1a) extracted from the dry tubers of *Rhizoma alismatis*. Polycyclic triterpene compounds have been demonstrated to be P-gp inhibitors [19,20]. Meanwhile, the structure of ALI is similar to that of alisol B 23-acetate (Figure 1b), which has been reported previously [21,22]. There is a possibility that ALI is a P-gp inhibitor with a pentacyclic structure. Herein we explore its ability to reverse P-gp-mediated MDR.

As Caco-2 cell monolayers overexpress P-gp after cultivation, they are often introduced as a model to screen P-gp inhibitors. In the article, this model was designed to investigate whether ALI was a P-gp inhibitor and, if so, a series of cell-based experiments were conducted using High Content Analysis (HCA). Doxorubicin is a potent antibiotic commonly used in human breast cancer chemotherapy. Doxorubicin has been chosen both as an anticancer drug and a P-gp fluorescence substrate. The direct inhibition of P-gp by ALI and its underlying mechanism were explored by studying the intracellular accumulation and nucleus distribution of doxorubicin in the presence of ALI. We also investigated the pharmacological function (early apoptosis) of doxorubicin to further confirm above results. Our research helped to elucidate the synergic effects of ALI with some anticancer drugs, as a part of identifying efficient and safe P-gp modulators from Traditional Chinese medicine. It is a promising contemporary strategy to enhance the efficacy of chemotherapeutic agents, especially anticancer drugs, with adjunctive therapy or alternative medication.

## 2. Results

### 2.1. The P-gp Inhibitor Probability and Reliability of Alisol F 24-Acetate

We predicted the P-gp inhibition of alisol B 23 acetate and alisol F 24 acetate using an ADME/Tox software (Percepta, ACD, Toronto, ON, Canada). The P-gp inhibitor probability and reliability of alisol F 24 acetate is stronger than that of alisol B 23 acetate as shown in Table 1.

**Table 1:** The P-gp inhibitor probability and reliability.

Compound Name	Probability	Reliability
Alisol B 23 acetate	0.49	0.85
Alisol F 24 acetate	0.89	0.81

### 2.2. Cell Viability of Caco-2 Cells Following Treatment with ALI

**Figure 2:**
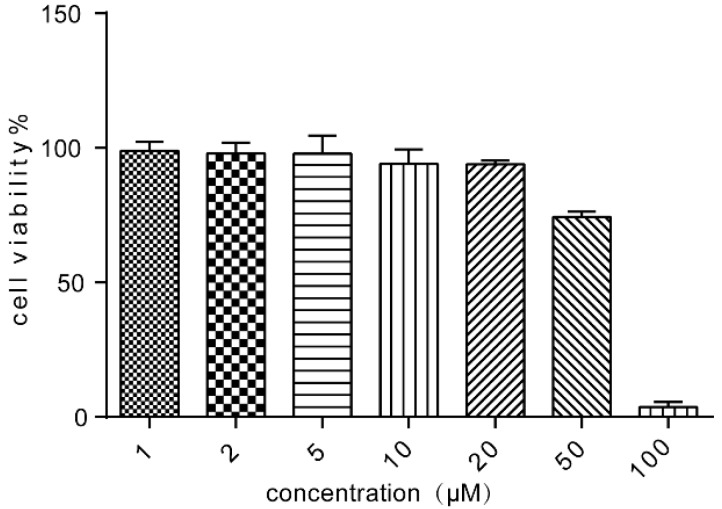
Cell viability of various concentrations of ALI on Caco-2 cells. Results were means ± SEM of three separate experiments.

To explore the toxicity of ALI to Caco-2 cells, various concentrations of ALI (1 μM–100 μM) were added to the cells for 24 h. As shown in Figure 2, ALI inhibited cell proliferation in a dose dependent manner. In our experiment, non-toxic concentrations of ALI (5 μM, 10 μM and 20 μM) causing cell growth inhibition lower than 20% were combined with doxorubicin in the reversal assays.

### 2.3. ALI Decreased the Efflux Ratio of Digoxin across Caco-2 Monolayers

**Figure 3:**
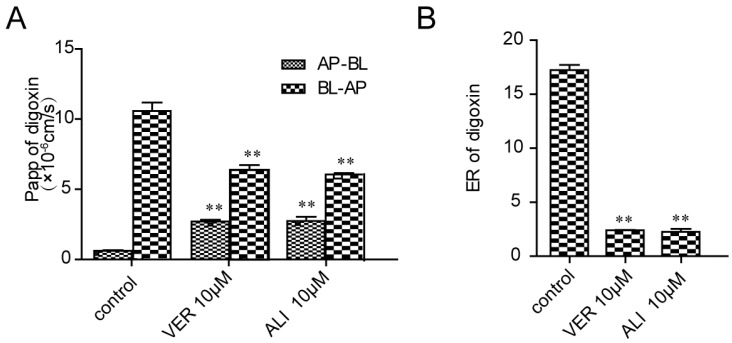
Effect of 10 μM ALI on the two-way transport of P-gp substrate digoxin across Caco-2 monolayers (**A**) Papp BL-AP and Papp AP-BL of digoxin; (**B**) the efflux ratio of digoxin. Results were means ± SEM of three separate experiments. Significance level ** *p* < 0.01.

Corresponding Papp values of digoxin in the absorptive (AP-BL) and secretory (BL-AP) directions and efflux ratio of digoxin are illustrated in Figure 3. Results showed that Papp values of digoxin in the absorptive (AP-BL) and secretory (BL-AP) directions of digoxin were (0.62 ± 0.07) × 10^−6^ cm/s and (10.6 ± 1.04) × 10^−6^ cm/s. Digoxin exhibited highly polarized transport across Caco-2 cell monolayers with marked efflux ratio 17.2. While in the presence of ALI, the transport of digoxin decreased by 4.47-fold in the BL-AP direction, it increased by 1.75-fold in the AP-BL direction across Caco-2 monolayers. The addition of ALI decreased its efflux ratios (ER = 2.27) implying that ALI exhibited transport polarity of P-gp substrate digoxin.

### 2.4. Multidrug Resistance of MCF-7/DOX Cells

**Figure 4:**
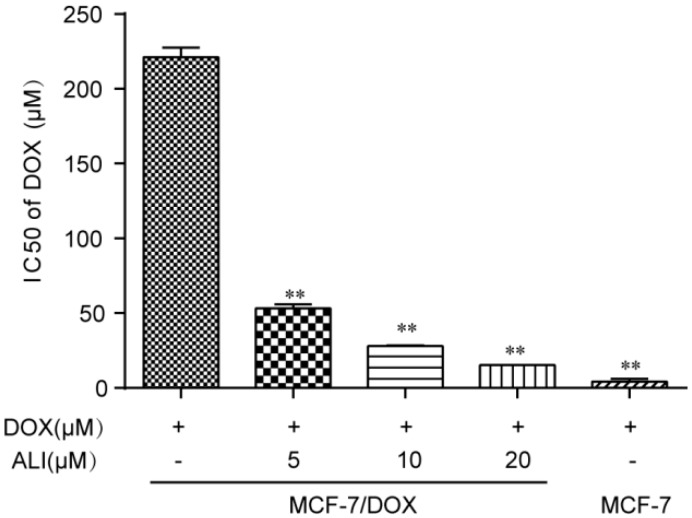
The effect of ALI on chemosensitivity and the effect of ALI on chemosensitivity of doxorubicin in MCF-7/DOX cells. MCF-7 and MCF-7/DOX cells were cultured for 24 h in the absence or presence of ALI (5 μM, 10 μM and 20 μM) with various concentrations of doxorubicin (0.03, 0.1, 0.3, 1, 3, 10, 30, and 100 μM). Data are presented as means ± SEM of triplicate determinations. Significance level ** *p* < 0.01.

To measure the multidrug resistance of MCF-7/DOX cells, various concentrations of DOX (0.03, 0.1, 0.3, 1, 3, 10, 30, and 100 μM) were added to the cells for 24 h. As can be determined from in Figure 4, the resistance index (RI) was 51.2, which indicated MCF-7/DOX cells were highly resistant to doxorubicin.

### 2.5. Cell Viability of MCF-7/DOX Cells Following Treatment with ALI

**Figure 5:**
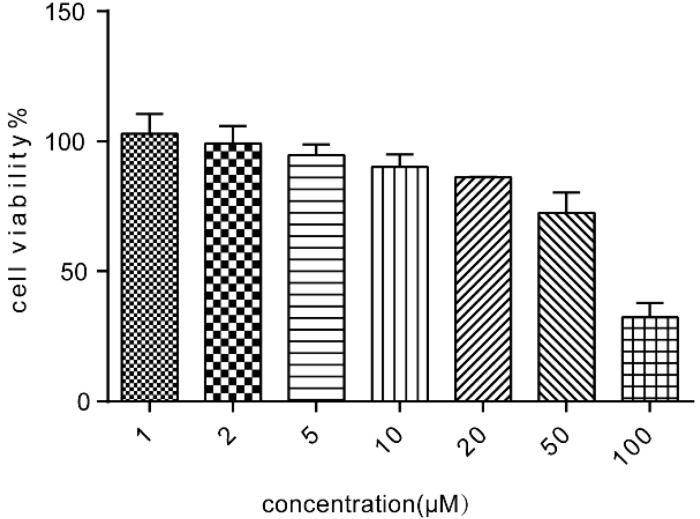
Cell viability of MCF-7/DOX cells following treatment with various concentrations of ALI. Results were means ± SEM of three separate experiments.

To determine the ALI toxicity on MCF-7/DOX cells, various concentrations of ALI (1 μM–100 μM) were incubated with cells for 24 h. Cell viability was evaluated by CCK-8 assay. As shown in Figure 5, ALI inhibited cell proliferation in a dose-dependent manner. For subsequent study, non-toxic concentrations of ALI (from 5 μM to 20 μM) with cell growth inhibition less than 20% were combined with doxorubicin.

### 2.6. ALI Enhanced Chemosensitivity of Doxorubicin in MCF-7/DOX Cells

Based on CCK-8 assay results, IC_50_ value of doxorubicin was apparently decreased in MCF-7/DOX cells when combined with 5 μM, 10 μM, and 20 μM ALI (Figure 4). Therefore, ALI significantly enhanced chemosensitivity of doxorubicin in a concentration-dependent manner.

### 2.7. The Synergic Activity of ALI in Combination with Doxorubicin

**Figure 6:**
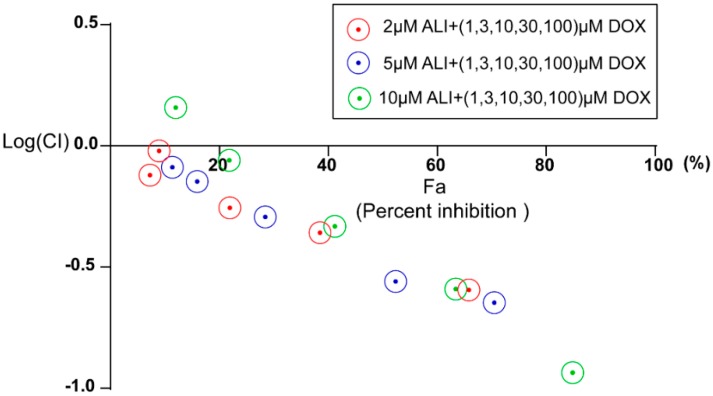
Combination index of different cell inhibition rate. Fa, the abbreviation of fraction affected, serves as the percent cell inhibition and CI represents combination index. The concentrations used for doxorubicin was 1, 3, 10, 30, 100 μM and that of ALI were 2, 5, 10 μM.

As shown in Figure 6, the majority of Log (CI) values were below zero, indicating that ALI has a good synergic activity with doxorubicin.

### 2.8. ALI Significantly Increased Intracellular Accumulation and Nuclear Migration of Doxorubicin in MCF-7/DOX Cells

**Figure 7:**
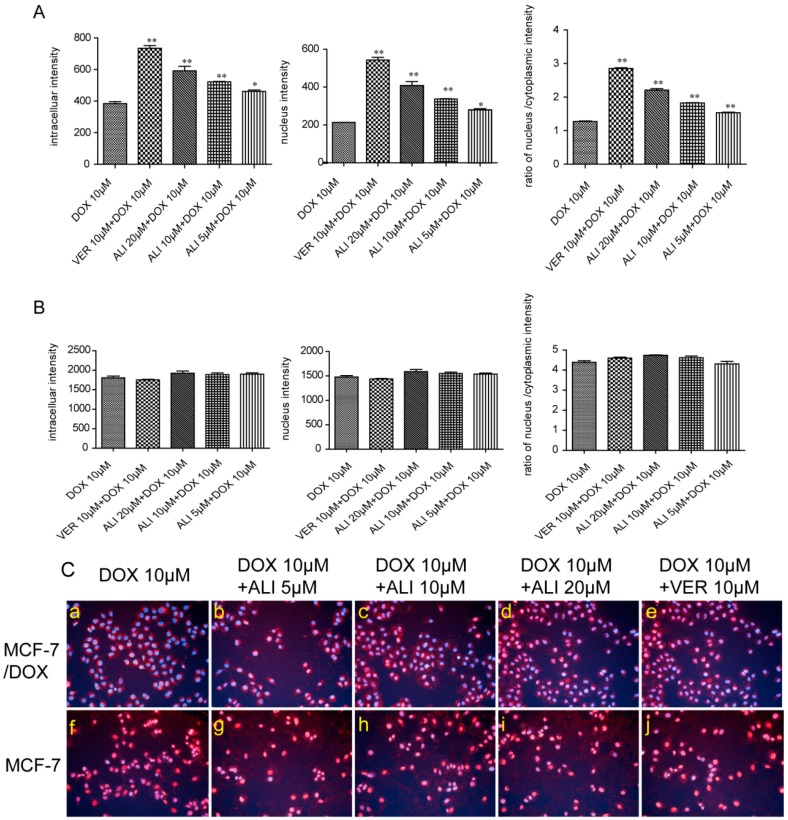
Influence of ALI on the accumulation and nucleus distribution of DOX in MCF-7/DOX cells and MCF-7 cells. (**A**) Influence of ALI on the accumulation and nucleus distribution of DOX in MCF-7/DOX cells; (**B**) Influence of ALI on the accumulation and nucleus distribution of DOX in MCF-7 cells. Data are presented as means ± SEM of triplicate determinations. Significance levels * *p* < 0.05; ** *p* < 0.01; (**C**) Influence of ALI on the nucleus distribution of DOX in MCF-7/DOX cells ((**a**) DOX 10 μM; (**b**) ALI 5 μM + DOX 10 μM; (**c**) ALI 10 μM + DOX 10 μM; (**d**) ALI 20 μM + DOX 10 μM; (**e**) VER 10 μM + DOX 10 μM) and MCF-7 cells (**f**) DOX 10 μM; (**g**) ALI 5 μM + DOX 10 μM; (**h**) ALI 10 μM + DOX 10 μM; (**i**) ALI 20 μM + DOX 10 μM; (**j**) VER 10 μM + DOX 10 μM) by HCA, magnification of representative images = 20×, red and blue fluorescence represent doxorubicin and nuclei. Purple fluorescence is an overlay of red and blue fluorescence).

As shown in Figure 7A,B, fluorescence intensity of doxorubicin of MCF-7 cells was 4.70-fold higher than that of MCF-7/DOX cells. In another words, the intracellular accumulation of doxorubicin in sensitive cells was 4.7 times the amount of that in MDR cells. When cells were treated with 5, 10, and 20 μM ALI, intracellular accumulation of doxorubicin in MCF-7/DOX cells increased by 1.20, 1.36, and 1.54-fold in a concentration-dependent manner (Figure 7A). Meanwhile, the effect of 20 μM ALI was just a little weaker than that of 10 μM positive drug verapamil. Neither verapamil nor ALI at various concentrations changed intracellular accumulation of doxorubicin in MCF-7 cells (Figure 7B).

Similar to its effect on intracellular accumulation, ALI significantly increased nuclear migration of doxorubicin in MCF-7/DOX cells in a concentration-dependent manner (Figure 7A) while had no effect on MCF-7 cells (Figure 7B).

As shown in Figure 7C, doxorubicin (labeled by red fluorescence) was distributed in the cytoplasm without gathering in the nuclei (labeled in blue by Hoechst33342) in untreated MCF-7/DOX cells. Addition of 5, 10, and 20 μM ALI or 10 μM verapamil not only increased doxorubicin intensity but also its migration into the nucleus (reflected in a color shift from blue to purple in nuclei, Figure 7C: a–e). In contrast, doxorubicin is mainly distributed in the nuclei (indicated by red and blue fluorescence overlay) in untreated MCF-7 cells (Figure 7C: f–j). All these results suggest that ALI not only increase intracellular accumulation of doxorubicin but also induce its migration into nuclei in MCF-7/DOX cells, but not MCF-7 cells.

According to semi-quantitative fluorescence assessment, the intracellular accumulation of doxorubicin in MCF-7 cells is 4.7 times the amount of that in MCF-7/DOX cells. As the concentration of ALI increases, the fluorescence intensity of doxorubicin in the nuclei gradually intensifies. Similarly, neither ALI nor verapamil have this effect on MCF-7 cells.

In data analysis, we select the ratio of fluorescence intensity in nucleus to fluorescence intensity in cytoplasmic (n/c ratio) to illustrate how ALI affected doxorubicin targeted distribution in cells. As shown in Figure 7, the n/c ratio in sensitive cells is nearly 3.5 times of that in MDR-cells. The n/c ratio is thus a more sensitive indicator for MDR phenomenon in MCF-7/DOX cells than intracellular accumulation and nuclear redistribution of doxorubicin in the nuclei.

### 2.9. ALI Promoted the Doxorubicin-Induced Early Apoptosis of MCF-7/DOX Cells in a Time-Dependent Manner

**Figure 8:**
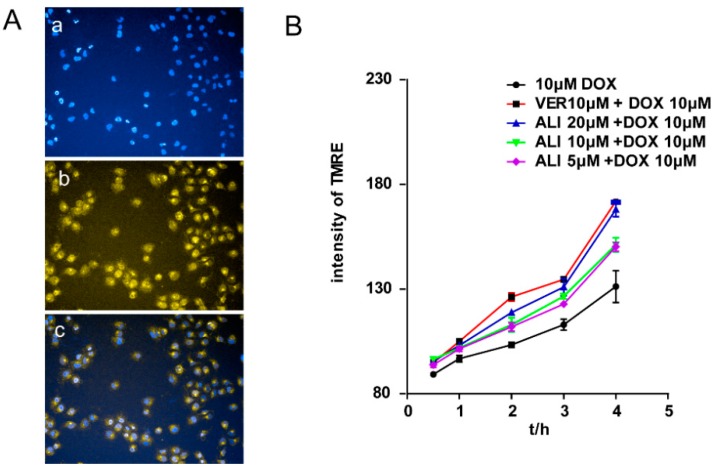
(**A**) Influence of 20 μM ALI on doxorubicin-induced early apoptosis of MCF-7/DOX cells at 2 h by HCA, magnification of representative images = 20×, orange and blue fluorescence represent TMRE and Hoechst33342. ((**a**) bule fluorescence of Hoechst3342 represents the location of nucleus; (**b**)orange flurescence of TMRE represents the level of mitochondrial transmembrane potential; (**c**) The merge represents doxorubicin-induced early apoptosis of MCF-7/DOX cells.) and influence of ALI on doxorubicin-induced early apoptosis of MCF-7/DOX cells at 0.5 h, 1 h, 2 h, 3 h, and 4 h. MCF-7/DOX cells were treated with doxorubicin or doxorubicin plus various concentrations of ALI for 0.5 h, 1 h, 2 h, 3 h, and 4 h. Data are presented as means ± SEM of triplicate determinations (**B**).

Cytoplasmic TMRE fluorescence intensity and mitochondrial membrane potential were used to monitor early apoptosis at 0.5 h, 1 h, 2 h, 3 h, and 4 h. When cells were treated with doxorubicin in the presence of various concentrations of ALI, TMRE fluorescence intensity was enhanced and mitochondrial membrane potential was decreased (Figure 8). Results showed that ALI promoted the doxorubicin-induced apoptosis of MCF-7/DOX cells in a time-dependent manner and concentration-dependent manner.

## 3. Discussion

In this study, ALI serves as a potent MDR-reversal inhibitor overcoming the multidrug resistance in MCF-7/DOX cells. Human breast cancer chemotherapy faces serious challenges in reversing MDR. Inhibition of the expression of P-gp will enhance chemosensitivity of MDR-cells. It has been reported that P-gp has several physiological effects including protection against toxic xenobiotics by excreting them into bile and urine, maintenance of the blood-brain barrier [23], and the transport of steroid hormones and cholesterol [24]. Many reversal agents from traditional Chinese medicine [25,26,27] have been investigated based on their inhibitory activity of ABC transporters. However, the potential of ALI reversing P-gp mediated MDR was demonstrated for the first time in this experiment.

In 2004, Fong *et al.*, [21] reported that alisol B 23 acetate could be a P-gp substrate. Additionally, alisol B 23 acetate was also a partial non-competitive inhibitor of P-gp. Their results suggested that alisol B 23 acetate might be a potent MDR reversal agent. Continued progress was made in 2007 by the same group that showed extract of the rhizomes of *Rhizoma Alismatis* had a synergistic growth inhibitory effect with cancer drugs that are P-gp substrates, including actinomycin D, puromycin, paclitaxel, vinblastine and doxorubicin [22]. However, the compound(s) that substantially contributed to the reversal effects were not revealed. Using an ADME/Tox software (Percepta, ACD), we predicted P-gp inhibition of alisol B 23 acetate and alisol F 24 acetate. The P-gp inhibitor probability of alisol F 24 acetate is stronger than that of alisol B 23 acetate. Given that prediction result, we put forward a supposition that alisol F 24 acetate may serve as a potent inhibitor suppressing P-gp activity in the extract of *Rhizoma Alismatis*. We have carried out the subsequent study to demonstrate that ALI is a P-gp inhibitor.

Caco-2 cell monolayers, originated from a human colonic adenocarcinoma cell line, overexpressed P-gp in the apical side after cultivation on polyester transwell plates for 21 days [28]. Therefore, it can be used as a model to screen P-gp inhibitors. To verify the hypothesis that ALI was a P-gp inhibitor, we established Caco-2 cell monolayers according to the requirements of the FDA guidelines. Dense polarized monolayer cells start to emerge after Caco-2 cells are seeded on a permeable polyester insert in 12 well culture plates for 21-day growth. At that time, P-gp is highly expressed in absorptive direction [29]. It would be easier for digoxin to transport from basolateral side to apical side. Polarity transport takes shape, which is presented in efflux ratio [30]. Addition of P-gp inhibitor weakens polarized barrier by narrowing the difference between apical and basolateral sides. As the two-way transport experiment provides a real simulation for drugs penetrating through membranes, Caco-2 cell monolayers are the golden model to evaluate the bonds between P-gp substrate and P-gp inhibitors [31,32]. Based on this model, digoxin with a high efflux ratio (ER = 17.2) was selected as a P-gp substrate indicating the Caco-2 monolayer model are reliable. Then the efflux ratio drops to 2.27 in the presence of ALI. What we have done succeeds in proving the former hypothesis that ALI serves as a potential P-gp inhibitor.

We need to further assess the inhibition level of ALI through MCF-7 cells and MCF-7/DOX cells. Verapamil was taken as the positive drug. It has once been used as a P-gp reversal agent, but unfortunately severely side effects have made it limited access to clinical treatment [11]. Doxorubicin is a chemotherapeutic drug for treatment of various kinds of tumors including breast cancer. Its target is the cell nucleus [33]. Non-toxic concentrations of ALI combined with some other anti-tumor drugs give a strong boost to the efficacy. In Caco-2 monolayer model, we have confirmed that ALI is a P-gp inhibitor. Then we study whether non-toxic concentrations of ALI enhance chemosensitivity of doxorubicin. We firstly investigate the efficacy of ALI in combination with doxorubicin in human breast MCF-7/DOX cells in an attempt to understand the reversal activity. IC_50_ value of doxorubicin apparently decreased in MCF-7/DOX cells when combined with ALI (5, 10 or 20 μM). The decrease of IC_50_ value confirms that ALI has the ability to reverse drug resistance and enhance chemosensitivity of doxorubicin in MCF-7/DOX cells. Meanwhile, the intracellular accumulation of doxorubicin in sensitive cells and MDR-cells will show a big difference. ALI can achieve success in reducing this difference. Then we design accumulation experiment in MCF-7/DOX cells and MCF-7 cells by HCA. Our data shows that the intracellular accumulation of doxorubicin in MCF-7 cells was 4.7 times the amount of that in MCF-7/DOX cells. Add of ALI increases intracellular accumulation of doxorubicin in MCF-7/DOX cells while it has no effect on MCF-7 cells. All this analysis has testified our previous hypothesis. It is the decreasing accumulation that causes drug resistance. As a result, ALI enhances the chemosensitivity of MCF-7/DOX cells.

As the target of doxorubicin is located in nucleus, the distribution of doxorubicin in cell will influence its efficacy [34]. The results of HCA demonstrated that doxorubicin mainly distributed in the nuclei in MCF-7 cells while majorly distributed in the cytoplasm in MCF-7/DOX cells. In the presence of ALI, the doxorubicin remarkably migrated from cytoplasm into nuclei in the MCF-7/DOX cells while no effect was seen in the sensitive cells. Meanwhile, the assays of the fluorescence intensity of doxorubicin in the nuclei and nucleus-cytoplasmic ratio also suggest the fact. The results further reveal that the resistance of MCF-7/DOX cells was deeply related to intracellular targeting distribution. ALI overcomes MDR by inducing the targeting distribution of doxorubicin in nuclei in MCF-7/DOX cells.

Doxorubicin plays the role in anticancer process by embedding DNA bases and inhibiting the synthesis of nucleic acids and the target of nuclear distribution is directly related to its efficacy. We examined the influence of ALI on doxorubicin-induced early apoptosis of MCF-7/DOX cells. Cell apoptosis was one of the important efficacy evaluation indexes of antitumor drugs. The phenomenon was first discovered by Kerr in 1972 [35]. The destruction of the mitochondrial trans-membrane potential that occurred before nucleus apoptosis (chromatin condensation and DNA fraction) is considered to be one of the earliest events in the cascade reaction of cell apoptosis [36]. Tetramethylrhodamine ethyl ester (TMRE), a membrane-permeable cationic fluorescent dye, can migrate into the mitochondrial matrix driven by the mitochondrial transmembrane potential. In normal cells, the fluorescence intensity becomes weaken or disappears. While cell apoptosis causes damage to the integrity of mitochondrial membrane and the collapse of the mitochondrial transmembrane potential, TMRE released the mitochondria emitting a strong orange fluorescence. Results showed that ALI dramatically promoted the doxorubicin-induced early apoptosis of MCF-7/DOX cells in a time-dependent manner.

In general, the reversal of P-gp can be achieved either by inhibiting the efflux function of P-gp or ATPase activity or regulating P-gp expression. Based on the above results, further experiments were needed to examine the effect of ALI on P-gp expression (either the transcriptional or protein level). Meanwhile, which compound really works and which one is more potent in the extract of Rhizoma Alismatis are still a hard and long way ahead of us.

In conclusion, ALI remarkably decreased the transport of digoxin in the BL-AP direction but increased that in the AP-BL direction, and decreased efflux ratio of digoxin. Our results clearly demonstrated that ALI is a powerful P-gp inhibitor. ALI reverses P-gp-mediated MDR and enhances chemosensitivity of doxorubicin by dose-dependently increasing its intracellular doxorubicin uptake and inducing nuclear localization in MCF-7/DOX cells. Moreover, ALI promoted doxorubicin-induced early apoptosis of MCF-7/DOX cells in a time-dependent manner implying that ALI synergistically enhanced antitumor effect of doxorubicin. Our study suggests that ALI may serve as a promising MDR reversal agent and a potential adjuvant for clinical cancer therapy in combination with antitumor drugs such as doxorubicin.

## 4. Materials and Methods

### 4.1. Chemicals and Reagents

ALI was purified 98% (Science and Technology Co., Ltd., Tianjin, China). Doxorubicin (a P-gp substrate) and verapamil (a P-gp inhibitor, a powerful MDR reversing agents) were purchased from Mellon Biological Technology Co., Ltd. (Dalian, China). Digoxin and digitoxin were produced by National Institute for the Control of Pharmaceutical and Biological Products (Beijing, China). Hoechst33342, rhodamine123 and tetramethylrhodamine ethyl ester (TMRE) were obtained from Introvigen (Waltham, MA, USA). Roswell Park Memorial Institute 1640 (RPMI 1640), Minimum Essential Medium (MEM), fetal bovine serum, and trypsin, penicillin-streptomycin were provided by Gibco (Grand Island, NY, USA). The CCK-8 kit (code number CK04) was supplied by the Dojindo Institute of Chemistry (Kumamoto, Japan).

### 4.2. Cell Lines and Cell Culture

The Caco-2 cell line was purchased from the cell bank of Chinese Academy of Sciences (Shanghai, China). MCF-7 cells were supplied by the American Type Culture Collection (ATCC, Shanghai, China). MCF-7/DOX cell was provided by XinYu Biological Technology Co., Ltd., (Shanghai, China). Caco-2 cell line was cultured in MEM containing 20% fetal bovine serum and 100 U/mL penicillin and streptomycin. Both MCF-7 and MCF-7/DOX cell line were grown in RPMI1640 containing 10% fetal bovine serum and 100 U/mL penicillin and streptomycin. All cell lines were maintained at 37 °C in a humidified atmosphere containing 5% CO_2_ in an incubator. Cell culture medium was changed every other day and cells were passaged upon reaching 80%–90% confluence.

### 4.3. The Prediction of P-gp Inhibitor Probability

P-gp plays an important role in absorption and distrubution of drugs that are P-gp substates and therefore is related to multidrug resistance.The prediction in silico models for the classification of P-gp inhibitors has been attatched great importance in drug discovery and development [37]. While staying focused on the ability of ALI on inhibiting P-gp, an ADME/Tox software (Percepta, ACD) was used to predict the probability of alisol F 24 acetate.A higher number signifies a higher probability and reliability.

### 4.4. Cell Viability of Caco-2 Cells Following Treatment with ALI

The cells were plated in 96-well plates at the density of 1 × 10^5^ cells/mL. After 24 h incubation, various concentrations of ALI were added to the wells, and then the plates were further incubated at 37 °C for 24 h. The medium was replaced with fresh culture medium containing 10% CCK-8 solution and the plates were incubated again for 3 h at 37 °C in dark. The absorbance (OD) of each well was measured at a wavelength of 450 nm with a FlexStation3.0 (Thermo Molecular Device, Waltham, MA, USA) and the cell viability was calculated using the following Equation (1):

(1) Cell viability % = (OD_ALI_ − OD_blank_)/(OD_control_ − OD_blank_) × 100%

### 4.5. Inhibition of P-gp Substrate Digoxin Transport in Caco-2 Cell Monolayers Following Treatment with ALI

HCA is a technology platform designed to acquire simultaneous determination for multiple targets and multiple parameters of cells following treatment with bioactive compounds. Imaging fluorescence microscopy serves as a rapid and instant tool for obtaining biological activity information from cells. In our study, it has focused on effectively and simultaneously evaluation on intracellular accumulation and nucleus distribution of doxorubicin in qualitative and semi-quantitative manners [38].

#### 4.5.1. Procedures of Caco-2-Monolayer Model

Caco-2 cells with a density of 1 × 10^5^ cells/mL were seeded on a permeable polyester insert (corning cell culture inserts, 0.4 μm pore size, 12 mm diameter; Corning Corporation, Corning, NY, USA) in 12 well culture plates and were used for the 21-day experiment. Measurements of transepithelial electrical resistance (Millicell-ERS epithelial volt-ohm meter; Millipore Corporation, Darmstadt, Germany) were used to evaluate the integrity of Caco-2 cell monolayers. The monolayers whose transepithelial electrical resistance values exceed 600 Ω·cm^2^ were used in transport studies. Before initiation of transport studies, the cell monolayers were first washed with warm HBSS (pH 7.4) twice and HBSS was preincubated in 37 °C on the shaking bed for the third wash. HBSS containing 10 μM ALI, 10 μM verapamil or medium were then loaded into both apical and basolateral chambers respectively. After 1.5 h incubationat 37 °C, 5 μM digoxin was added to either the apical or basolateral side to evaluate the transport in absorptive and secretory directions, and the cell monolayers were incubated for another 2 h. At the designated time point, samples were taken from the receiving chamber for analysis. Concentration of digoxin was determined by LC-MS/MS.

#### 4.5.2. Analysis of Digoxin in HBSS Buffer Samples

An aliquot of 100 µL of the sample with 10 µL of internal standard (IS, digitoxin) spiked were extracted by 1 mL ethyl acetate. After 10 min centrifugation, 900 µL of the extracted organic layer was evaporated to dryness with a SpeedVac system (Eppendorf, Hamburg, Germany). The residue was reconstituted in 100 µL of 50% acetonitrile, followed by 5 min centrifugation. Then 2 µL of the supernatant was injected for LC-MS/MS analysis. The system consisted of UPLC system (Waters, Milford, MA, USA) and triple quadrupole tandem mass system (Waters) equipped with an electrospray ionization source. The analytical column was a BEH C18 column (2.1 × 50 mm, 1.7 μm, ACQUITY, Waters) with a flow rate of 0.3 mL/min. The column and autosampler tray temperatures were 40 °C and 4 °C respectively. Gradient elution was performed with 0.1% formic acid solution (eluent A) and acetonitrile (eluent B): 0–0.30 min, 5% B; 0.30–0.31 min, 5%–20% B; 0.31–1.00 min, 20%–30% B; 1.00–2.00 min, 30%–40% B; 2.00–2.30 min, 40%–95% B; 2.30–3.00 min, 95% B; 3.01–3.5 min, 5% B. Mass spectrometer was operated in the negative ionization mode. Nitrogen was used as dry gas. Quantitation was performed using MRM of the transitions of *m*/*z* 779.48→649.48 for digoxin and *m*/*z* 763.54→633.48 for the IS. Data acquisition was performed with Masslynx 4.1 software (Waters).

#### 4.5.3. Data Analysis

For the transport assay, the apparent permeability coefficient (Papp) is presented in centimeters per second and calculated as in Equation (2):

(2) Papp = (ΔQ/Δt)/(A × C_0_)

where ΔQ/Δt is the permeability rate (nanogram per second), A is the surface area of the membrane (centimeters squared); and C_0_ is the initial concentration in the donor chamber (nanomolar).

The efflux ratio (ER) was calculated as in Equation (3):

(3) ER = Papp(BL − AP)/Papp(AP − BL)

### 4.6. Multidrug Resistance of MCF-7/DOX Cells

It was a similar method to Caco-2 cells for MCF-7 and MCF-7/DOX cells. The concentrations required to inhibit growth by 50% (IC_50_ values) of DOX on the two cell lines were calculated by GraphPad Prism 5.0 and resistance index (RI) was calculated using the following Equation (4):

(4) RI = IC_50_ of doxorubicin on MCF-7/DOX cells/IC_50_ of doxorubicin on MCF-7 cells

### 4.7. Cell Viability of MCF-7/DOX Cells Following Treatment with ALI

It was a similar method to Caco-2 cells for MCF-7 and MCF-7/DOX cells. The cell viability was calculated using the following Equation (5):

(5) Cell viability % = (OD_ALI_ − OD_blank_)/(OD_control_ − OD_blank_) × 100%

### 4.8. MDR Reversal Activity of ALI

The MCF-7/DOX cells were plated in 96-well plates at a density of 1 × 10^5^ cells/mL, and the experiment was determined by the CCK-8 kit. After 24 h incubation, the wells were divided into two groups. One group was treated with various concentrations of doxorubicin alone while the other was treated with various concentrations of doxorubicin combined with ALI (5 μM, 10 μM, and 20 μM) for 24 h at 37 °C. The medium was replaced with fresh culture medium containing 10% CCK-8 solution and the plates were incubated for a further 3 h at 37 °C in dark. The absorbance of each well was measured at a wavelength of 450 nm with a FlexStation3.0 (Molecular Device). IC_50_ values of doxorubicin on the two cell lines were calculated by GraphPad Prism 5.0 (GraphPad Software, Inc., La Jolla, CA, USA) and fold reversal (FR) was calculated using the following Equation (6):

(6) FR = IC_50_ of DOX without ALI/IC_50_ of DOX in combination with ALI

In order to better assess the nature of the interaction between alisol F 24 acetate and doxorubicin, the CompuSyn 1.0 software [39] was used to plot the effect. The concentrations used for doxorubicin was 1, 3, 10, 30, 100 μM and that of ALI were 2, 5, 10 μM. A Combination Index (CI) less than, equal to and larger than 1 indicates synergism, additive effect, and antagonism, respectively. Based on these algorithms, computer software has been developed to allow automated simulation of synergism and antagonism at all dose or effect levels. Fa, the abbreviation of fraction affected, serves as the percent cell inhibition and CI represents combination index.If the percentage of cell inhibition (Fa) *vs.* Log (CI) < 0, it indicates that ALI has a good synergic activity with doxorubicin.

### 4.9. Intracellular Influx and Nuclear Distribution of DOX in MCF-7/DOX Cells Following Treatment with ALI

The MCF-7/DOX cells were plated in 96-well plates at a density of 1 × 10^5^ cells/mL. Five treatment groups were designed: negative control with medium only, positive control with 10 μM verapamil, test groups with 3 different concentrations of ALI (5 μM, 10 μM, and 20 μM). After 2 h incubation at 37 °C, 10 μM doxorubicin was added and the incubation continued for another 2 h. The drugs were removed rapidly and cells were washed once with ice-cold phosphate-buffered saline (PBS) and fixed with 4% formaldehyde solution for 10 min in dark. After a PBS wash, cells were treated with 1 μg/mL Hoechst33342 (nucleus dye) for 20 min in dark. The dye was removed quickly and wells were washed three times with PBS. The fluorescence intensity of the intracellular doxorubicin and the nuclear doxorubicin were detected by High Content Analysis instrument (Operetta, PerkinElmer, Waltham, MA, USA) and their ratios were calculated. Fluorescence excitation wavelength and emission wavelength of Hoechst33342 (blue light) and doxorubicin (red light) were Ex = 380 nm/Em = 445 nm and the Ex = 535 nm/Em = 595 nm respectively.

### 4.10. Determination of Doxorubicin-Induced Early Apoptosis in MCF-7/DOX Cells Following Treatment with ALI

The MCF-7/DOX cells were plated in 96-well plates at a density of 1 × 10^5^ cells/mL. The following treatment groups were designed: negative control with medium only, positive control with 10 μM verapamil combined with 10 μM doxorubicin, test groups with three different concentrations of ALI (5 μM, 10 μM, and 20 μM) combined with 10 μM doxorubicin. After 0.5 h, 1 h, 2 h, 3 h, and 4 h incubation at 37 °C, the drugs were removed rapidly and cells were washed once with ice-cold PBS and then treated with 1.5 μg/mL TMRE and 1 μg/mL Hoechst33342 for 30 min in dark. The dyes were absorbed quickly and then were washed three times with PBS. Cytoplasmic TMRE fluorescence intensity was detected by High Content Aanlysis (HCA) instrument (Operetta) and their rations were calculated. Fluorescence excitation wavelength and emission wavelength of Hoechst33342 (blue light) and TMRE (orange light) were Ex = 380 nm/Em = 445 nm and the Ex = 549 nm/Em = 574 nm respectively.

### 4.11. Statistical Analysis

All the values are expressed as the means ± the standard error of the mean (SEM). Statistical analysis was performed using GraphPad Prism 5.0. The significance of differences was determined using one-way analysis of variance (ANOVA) followed by Dunn’s test. A *p*-value <0.05 was considered statistically significant.
